# Efficacy of a combination of 10% imidacloprid and 4.5% flumethrin (Seresto®) in slow release collars to control ticks and fleas in highly infested dog communities

**DOI:** 10.1186/1756-3305-6-210

**Published:** 2013-07-18

**Authors:** Emanuele Brianti, Luigi Falsone, Ettore Napoli, Chiara Prudente, Gabriella Gaglio, Salvatore Giannetto

**Affiliations:** 1 Dipartimento di Scienze Veterinarie, Università degli Studi di Messina, Messina, Italy

**Keywords:** Imidacloprid/flumethrin, Ticks, Fleas, Treatment, Dog, Shelter, Flea allergic dermatitis

## Abstract

**Background:**

Dog communities living in kennels are at high risk of being infected by ticks and fleas. In spite of the indubitable efficacy of several topical ectoparasiticides registered for the control of ectoparasites in dogs, the short period of action and the price of these products limit their use in dog communities. This paper reports on the efficacy of imidacloprid/flumethrin slow release collars to cure dogs highly infested with ectoparasites and to prevent infestations for 8 months in a refuge with a history of unsuccessful environmental treatments.

**Methods:**

A total of 82 dogs were collared with slow release collars containing a formulation of imidacloprid 10% / flumethrin 4.5%. Dogs were followed-up for ectoparasite presence after 2, 7 and 14 days and thereafter biweekly up to 90 days. Furthermore, dogs were examined for ectoparasites whilst replacing collars 8 months after their application.

**Results:**

At the time of treatment 79 (96.3%) out of 82 included dogs were heavily infested by ticks and 53 (68.8%) out of the 77 combed dogs were infested by fleas. Tick infested dogs had an estimated mean intensity of 46.9 (± 65.7), while flea infested animals had a load between 20 and 50 fleas. In addition, some of the flea infested dogs (18.9%) were presenting signs of flea allergic dermatitis (FAD). Two days after treatment, 49 (60.5%) and 9 (11.7%) dogs were still infested by live ticks and fleas, respectively. However, the mean intensity of ticks decreased to 3.5 (± 4.3) with a reduction of 92.5%. Except for sporadic cases, no attached ectoparasites were found on dogs from the day 14 visit until the end of the investigation. Cases of FAD resolved without any other treatment within 30 days.

**Conclusions:**

Ticks and fleas represent a constant hazard for dog populations. Therefore, in particular settings, such as dog refuges, sustainable and long-term strategies to control ectoparasite infestations are needed. Based on the observed evidence of efficacy, long-term duration and safety, the imidacloprid/flumethrin slow release collars can be regarded as an efficacious and sustainable means for ectoparasite control and for treatment of FAD in high-risk dog communities.

## Background

Ticks and fleas represent a major concern for dogs for their primary injury on infested animals and for their role as vectors of virus, bacteria, protozoa and helminths [1-3]. Tick-borne diseases are indeed regarded among the principal causes of illness/death in endemic areas; additionally, some of the main tick-borne transmitted pathogens (e.g. *Ehrlichia*, *Babesia*, *Borrelia*, *Anaplasma* and *Rickettsia* species) are also zoonotic agents of great public health concern [3,4]. In southern Europe, ticks and fleas represent a year-round hazard, although in these areas, ticks are more active from March to October, during spring and summer seasons [5]. Ownerless dogs are still a sanitary and animal welfare issue in many European countries and it has been estimated that stray dogs account for about 75% of the global population of dogs [6]. In Italy, stray dogs are controlled by spaying campaigns and by hosting them in shelters until their adoption or for their lifetime [7]. Local authorities are in charge not only of their maintenance in shelters but also their sanitary and preventive sustenance. However, due to limited funds currently available, in many shelters dogs do not receive adequate sanitary care. For this reason, in spite of the efficacy of several topical ectoparasiticides registered for the control of ticks and fleas in dogs, the short period of action (about 28 days) and the price, limit their use in shelters or dog refuges. Therefore, biocide molecules (e.g. organophosphates or pyrethroids) are periodically sprayed, mostly off label, on dogs and pen surfaces during the summer seasons to control ectoparasites [8]. Nonetheless, the efficacy of this practice in controlling ectoparasites is unpredictably conditioned by numerous variables such as the concentration of molecules, the frequencies of spraying and their residual effect in the environment and on dogs [9]. In addition, toxicity for both personnel and animals represents a major concern for this strategy [10,11]. Recently, a combination of 10% imidacloprid and 4.5% flumethrin (Seresto®, Bayer Animal Health) has been developed for use in dogs and cats in a polymer matrix collar [12,13]. By virtue of a combined repellent and insecticidal activity against fleas and ticks and of the slow release collar matrix system, the protection against these ectparasites has been demonstrated over a period of eight months [13]. In addition, this combination has recently proved to confer long-term protection against infection by *Leishmania infantum* to dogs located in a hyper-endemic area of southern Italy [14]. This paper reports on the efficacy of imidacloprid/flumethrin slow release collars to cure dogs highly infested with ectoparasites and to prevent new infestations for 8 months in a refuge with a history of an unsuccessful series of environmental treatments with biocides. As a consequence of their high efficacy in controlling fleas, the collars also proved to be efficacious in curing cases of flea allergic dermatitis.

## Methods

### Site and environment

The investigation was conducted in a dog shelter in Lentini (N 37.284637°, E 14.995900°, province of Syracuse, Sicily, Italy). The shelter is a volunteer-based refuge for dogs where about one hundred ownerless dogs are housed in pens built on natural ground in a citruses plantation. Pens have a surface of approximately 20 m^2^ with a mean density of 2.7 dogs per pen. Tick and flea infestation was usually controlled by weekly spraying biocides (i.e. Amitraz, Taktik® 125, Farmaceutici Gellini s.r.l.) on dogs and on pen surfaces. However, this practice was not efficacious in controlling ectoparasites. Indeed, despite frequent environmental treatments, the veterinarians responsible for the shelter complained about the massive tick and flea infestation of dogs during the months of June and July 2012. The ectoparasite infestation was so high that it induced reluctance in volunteers to care for these animals as they were scared to go through infested pens.

### Animals and procedures

In August 2012 a total of 82 (51 females and 31 males) mongrel dogs from 6 months to 12 years-old and with a body weight ranging from 8 to 28 kg were collared at the same time with slow release collars containing a formulation of imidacloprid 10%/flumethrin 4.5% (Seresto®, Bayer Animal Health). Before placing the collars, tick load was evaluated by checking a total of 14 anatomical sites (i.e. face, ears (right and left), neck, forelimbs (right and left), armpits (right and left), thorax, abdomen, thighs (right and left) and hindlimbs (right and left)), and by sorting the number of attached ticks for each site examined into five classes, i.e. 1–5, 6–20, 21–50, 51–100, >100. Flea presence and abundance were respectively assessed by combing dogs tolerating this procedure for ~3 minutes and by sorting them into three classes, i.e. 1–5, 6–20, 21–50. Dogs were identified using their microchip code, and for each of them a clinical form, including the dogs’ data, anamnestic information and ectoparasite estimation, was filled out at the time of treatment. Dogs were followed-up for ectoparasite presence after 2, 7 and 14 days and biweekly thereafter up to 90 days. Furthermore, dogs were examined again for ectoparasite presence at the replacement of collars that occurred 8 months (day 250) after their application. To avoid any bias, parasitological examinations and replacement of collars were performed by the same operator for all control visits. Biocide treatments had been stopped 10 days before collar application and were not repeated anymore. Animals included in this investigation were not enrolled in any experimental trial and were managed according to local Italian animal welfare regulations. The collars were a registered product and were applied in accordance with the registered claims and following the manufacturers instructions. Samples of ticks (~50) and fleas (~50) were randomly collected from infested dogs at the time of treatment and during the following controls. These parasites were stored in vials containing 70% alcohol and subsequently identified at species level using morphometrical keys for ticks [15,16] and for fleas [17].

### Data analysis

Data collected in the clinical forms were transferred into an electronic spreadsheet (Excel® 2010, Microsoft) for data elaboration and analysis. To perform easier data management, classes of infestations estimated at the time of inclusion for each site and for each dog were converted to number, using the mean value of class or to “101” for the class “>100” as reported in the following: class 1–5 = 3; class 6–20 = 13; class 21–50 = 35; class 51–100 = 75; class >100 = 101. Ecological indexes, i.e. mean intensity (ratio between tick abundance and number of positive animals), mean abundance (ratio between tick abundance and number of examined animals), were calculated for ticks infesting dogs according to Bush *et al.*[18]. At the time of treatment, total tick abundance and frequency (ratio between the number of tick infested sites and the number of examined sites) were calculated for each examined site. Mean value of positive sites per dog at day 0 was also calculated to assess the pattern of infestation (e.g. clustered or scattered). Inverse-Distance-Weighting multivariate interpolation method (IDW) was used to interpolate data on tick infestation load (i.e. frequency and abundance) from the examined sites to the entire surface of dog body, considered for the purpose as bidimensional [19]. A dog map was generated and IDW was calculated and graphically represented using ArcMap^TM^ 10.0 (ESRI Inc., USA).

**Table 1:** Number and prevalence of tick infested dogs, and ecological index values for ticks in 82 dogs hosted in the shelter before (Day 0) and after (Day 2 – Day 250) the application of imidacloprid/flumethrin slow release collars

	**Visit**									
	**Day 0**	**Day 2**	**Day 7**	**Day 14**	**Day 30**	**Day 45**	**Day 60**	**Day 75**	**Day 90**	**Day 250**
Dogs (n)	82	81	80	79	78	78	77	77	77	77
Positive (n)	79	49	12	0	1	0	0	0	0	2
Prevalence (%)	96.3	60.5	15.0	0.0	1.3	0.0	0.0	0.0	0.0	2.6
Tick Mean Intensity (± St. Dev.)	46.9 (± 65.7)	3.5 (± 4.3)	1.8 (± 0.7)	0.0	2.0	0.0	0.0	0.0	0.0	1.0
Tick Mean Abundance (± St. Dev.)	45.2 (± 66.4)	2.1 (± 5.1)	0.3 (± 0.8)	0	0.3	0	0	0	0	2.6 (± 0.2)

## Results

**Figure 1:**
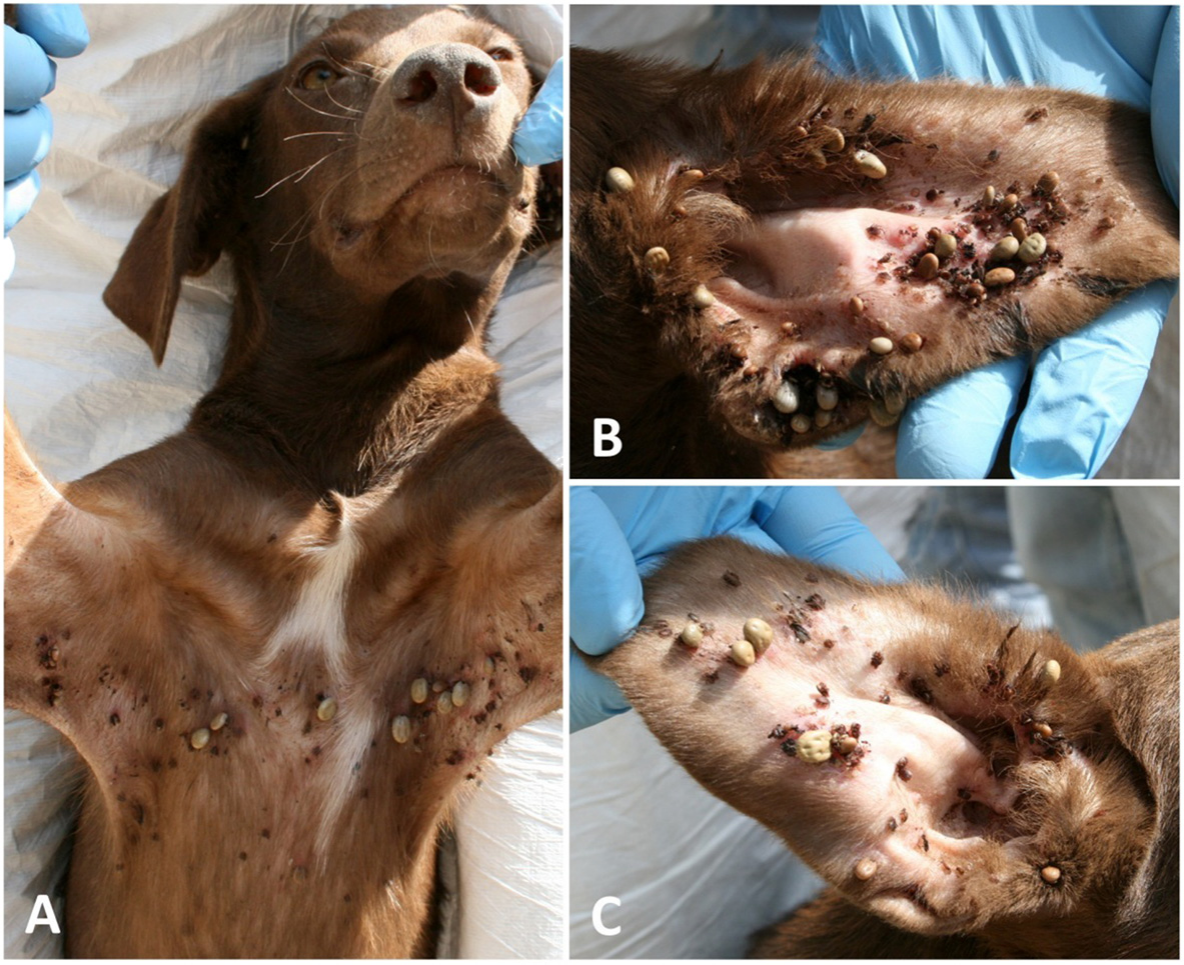
**Tick infestation in a dog before the application of collar (Day 0).** Tick abundance was higher on armpits, thorax **(A)** and ears **(B**-**C)**.

**Figure 2:**
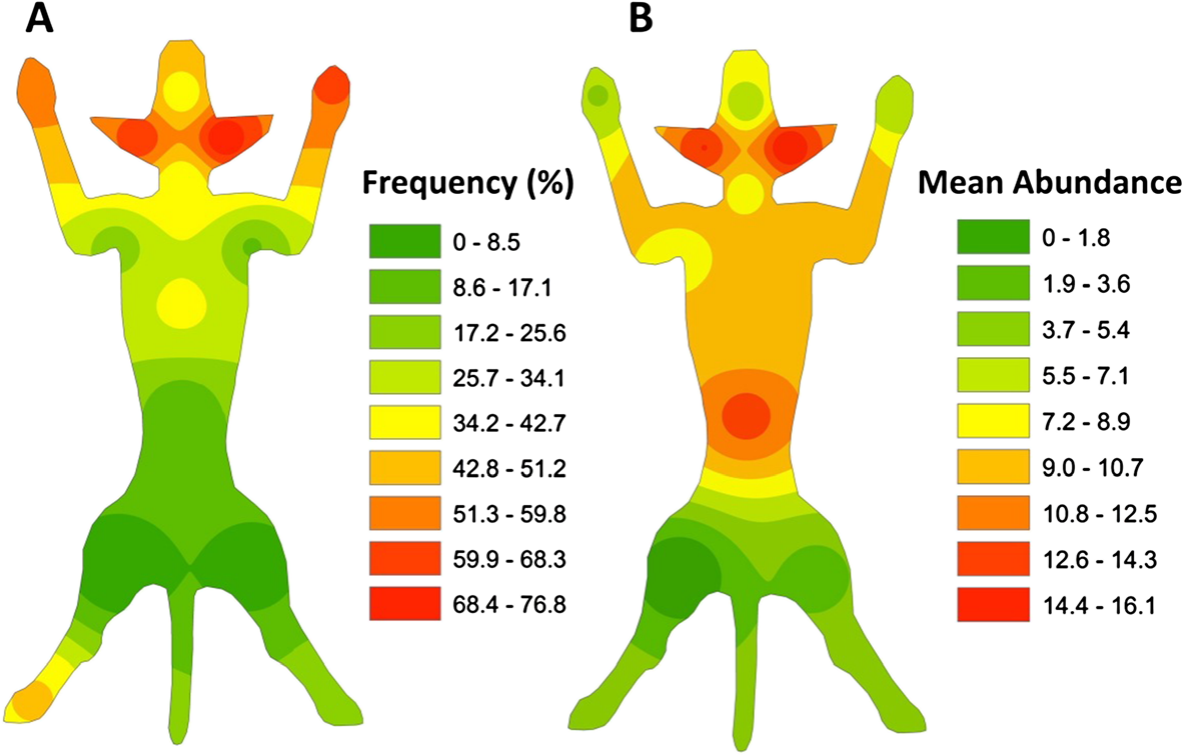
Frequency (A) and mean abundance (B) of ticks on the body of infested dogs estimated using Inverse Distance Weight algorithm of data collected in 14 anatomical sites.

**Figure 3:**
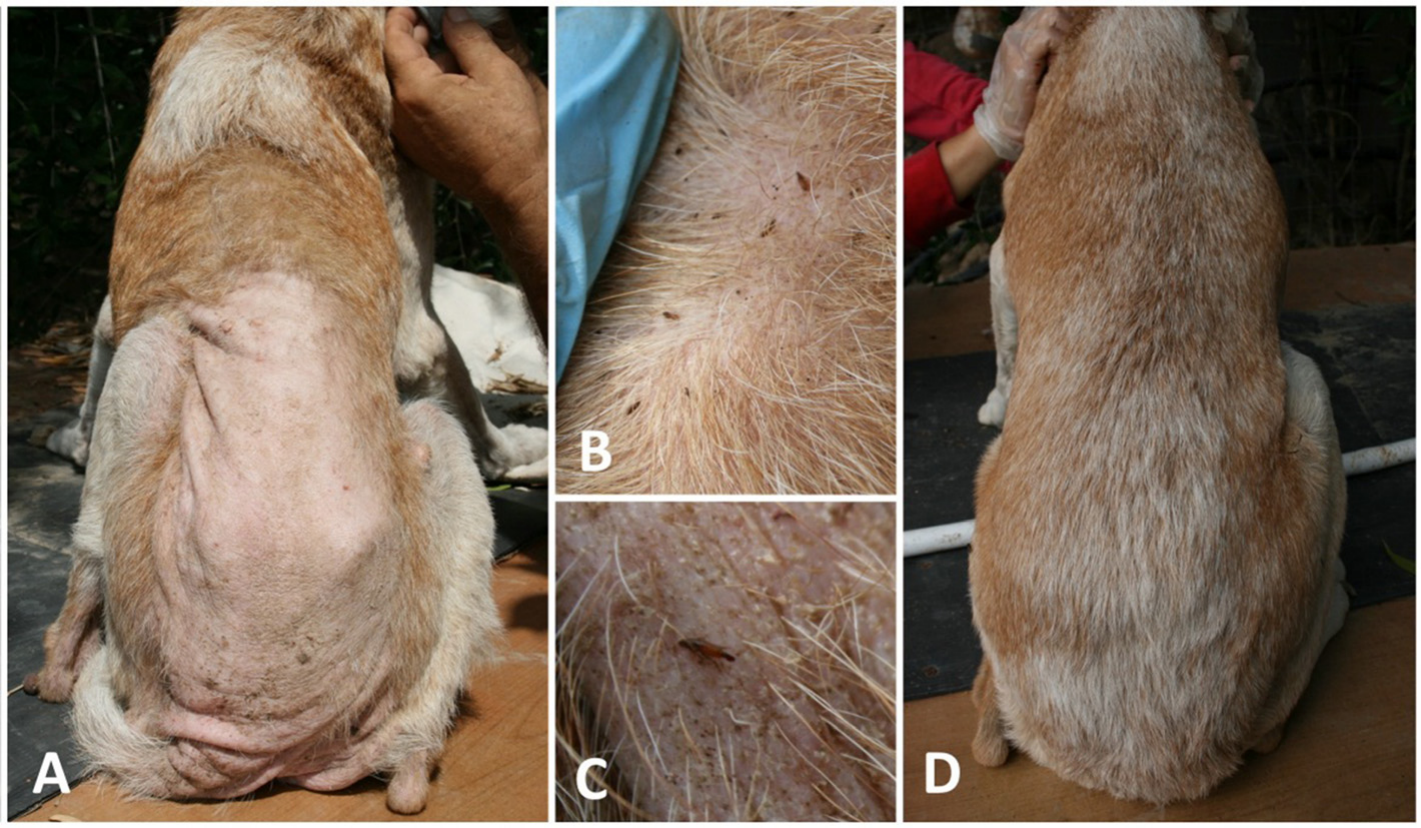
Dog presenting a severe flea allergic dermatitis (A) with a severe flea infestation (B-C); resolution of dermatitis and alopecia in the same dog 30 days after the application of collar (D).

**Figure 4:**
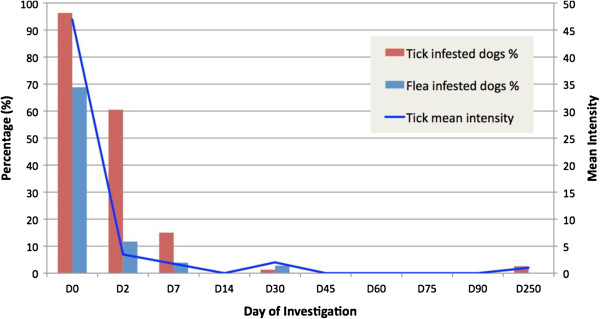
Percentage of ectoparasite infested dogs and tick mean intensity observed at the application of collars (D0) and in the follow-ups (D2-D250).

**Figure 5:**
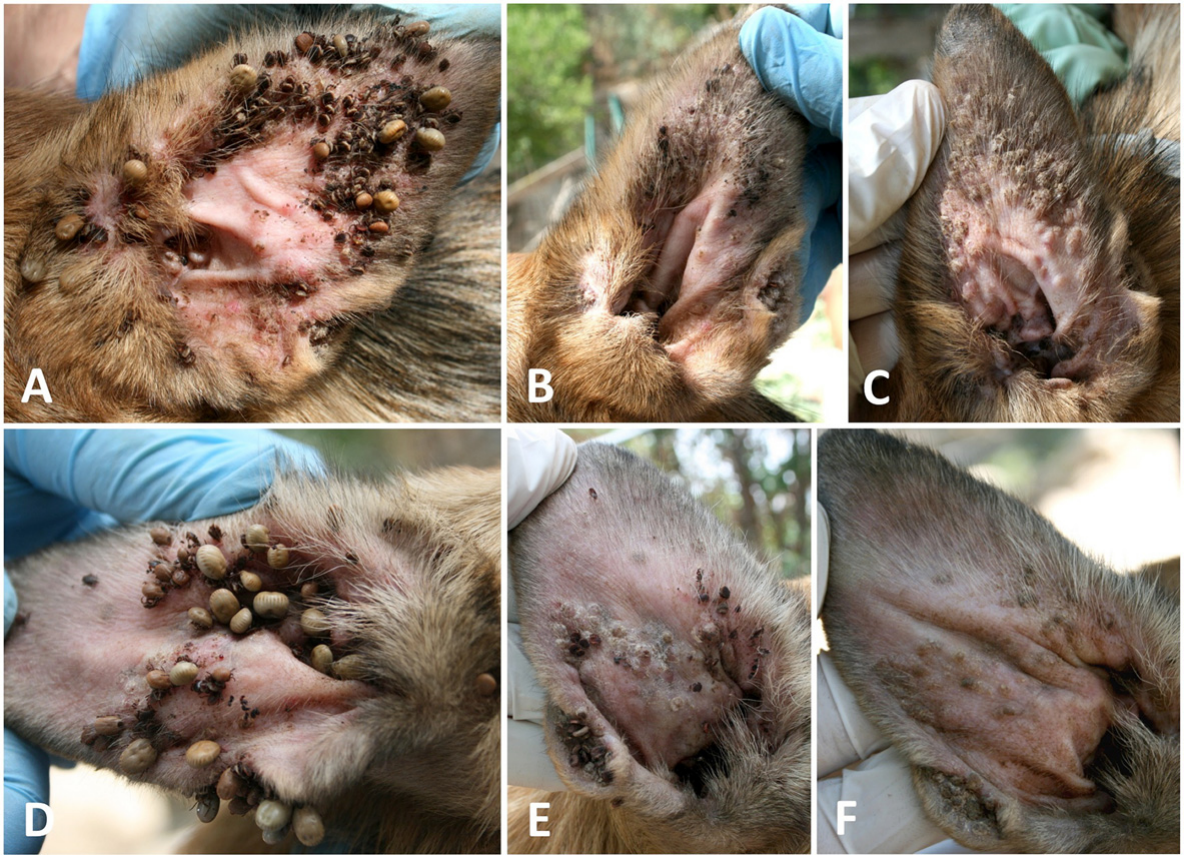
Cure of severe tick infestations in two dogs enrolled in the trial (A and D); Day 2 follow-up (B-E); Day 7 follow-up (C and F).

At the time of treatment, 79 (96.3%) out of 82 included dogs were infested by ticks. Tick infested dogs had a mean intensity of 46.9 (± 65.7) and a mean abundance of 45.2 (± 66.4) (Table 1); although about a quarter of the dogs (24.1%) were heavily infested ranging from 51 to 434 ticks per dog (Figure 1). Ticks were unevenly attached on the examined sites, with significantly more found on the ears (left 67.1%, right 76.8%), forelimbs (left 54.9%, right 61.0%) and hindlimbs (left 43.9%, right 42.7%), whereas the lowest infestation frequency was recorded in thighs (left 0.0%, right 3.7%) (χ^2^=168.21; *P* < 0.0001) (Figure 2A). Tick abundance was higher on ears, abdomen, neck and thorax (Figure 2B). The average number of tick infested sites per dog was 5.1. Of the 77 dogs that tolerated combing, 53 (68.8%) were infested by fleas, with an estimated load between 20–50. In addition, 18.9% (10) of flea-infested animals presented clinical conditions associated with flea allergic dermatitis (FAD) (Figure 3, Table 2). A total of 5 dogs were lost to follow-up. In particular, two dogs died at day 2 and day 7 with aggression and acute disease, respectively. Two other dogs were adopted at day 14 and at day 30, while one dog was un-collared at day 45 due to dermatitis on the ventral side of neck. Forty-eight hours after collar application, 49 (60.5%) and 9 (11.7%) dogs were still infested by live ticks and fleas, respectively. However, the mean intensity for ticks had decreased to 3.5 (± 4.3) with an overall reduction of 92.5% (Figures 4 and 5, Table 1). After seven days, the number of tick and flea infested dogs had decreased to 12 (15%) and 3 (3.9%), respectively. No attached ticks or fleas were found on dogs at the day 14 visit and no ectoparasites were found on dogs in the further controls, except one tick infested dog showing two nymphs in the inter-digital spaces of forelimbs and two flea infested dogs (class 1–5) observed at the day 30 visit (Table 1 and 2). After eight months, at the time of replacement of collars, only two (2.6%) dogs out of the remaining 77 animals were found to be infested by ticks while none of the dogs examined were infested by fleas. All cases of FAD resolved without any other treatment within 30 days after the application of collars (Figure 3). Collars were well tolerated and no side effects were observed with the exception of the one dog mentioned above that showed an erythematous dermatitis on the ventral side of neck at day 45 post treatment. The dog was un-collared and dermatitis resolved without any treatment within two weeks. The ticks collected from dogs for speciation were all identified as *Rhipicephalus sanguineus sensu alto*[17], while fleas were all consistent with the species *Ctenocephalides felis*.

**Table 2:** Number of flea infested dogs before (Day 0) and after (Day 2 – Day 250) the application of imidacloprid/flumethrin slow release collars

	**Visit**									
	**Day 0**	**Day 2**	**Day 7**	**Day 14**	**Day 30**	**Day 45**	**Day 60**	**Day 75**	**Day 90**	**Day 250**
**Positive**	53	9	3	0	2	0	0	0	0	0
**Negative**	24	68	74	74	72	74	74	74	74	74
**Examined**	77	77	77	74	74	74	74	74	74	74

## Discussion

**Figure 6:**
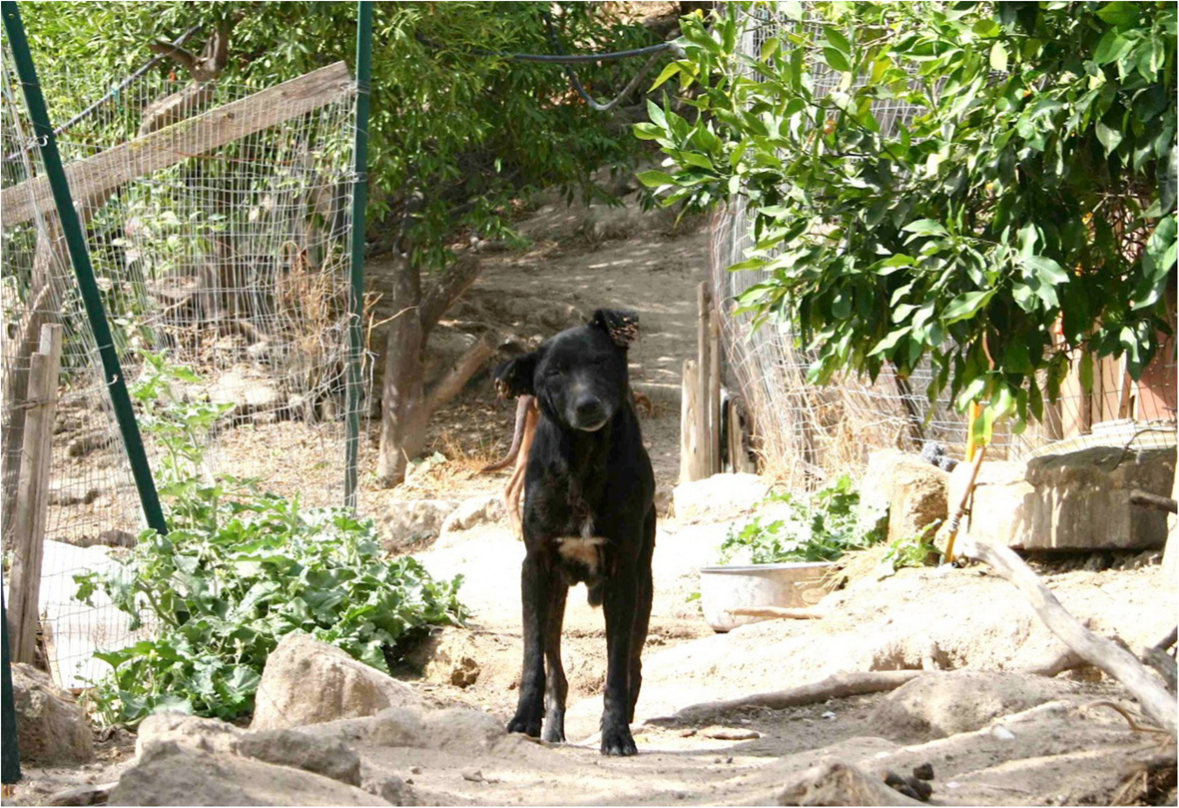
Un-collared dog allowed to free-range inside the shelter showing a severe tick infestation at day 60 of investigation.

The present investigation reports novel data on the efficacy and safety of the combination of imidacloprid/flumethrin in a polymer matrix slow release collar for the treatment and the prevention of tick and flea infestations in a high-risk exposed community of dogs. The combination proved to be safe and efficacious in rapidly curing pre-existing infestations and successfully preventing new ones over a period of 250 days. Additionally, the collar proved to be very effective for the treatment of FAD. Although numerous environmental treatments had been performed before collar application, the sanitary conditions of dogs at the beginning of the investigation appeared extremely dramatic. Indeed, most of the enrolled dogs were heavily infested by ticks and fleas with a considerable percentage of them clearly suffering from these conditions and/or presenting associated diseases such as FAD. In accordance with previous reports [5,20-22] the most frequent tick infested sites were located in the anterior regions of dogs (e.g. head, ears, neck and forelimbs). The evident difficulties of dogs in self-grooming on some of these sites to actively remove ticks could be accounted as one of the main explanations of the uneven distribution observed [22]. However, other reasons such as skin thickness, odours [5], and arrival point of questing stages [23] have also to be taken into account to explain this un-scattered distribution. The heavy tick infestation and parasitic pressure experienced by dogs enrolled in this investigation is evidenced by the value of mean intensity recorded at day 0 (46.9), which is two-fold higher than that recorded in a previous study conducted in a similar setting, season and dog population [5]. The scarce efficacy observed for the environmental treatments might be explained by numerous variables affecting the worth of this control measure [9]. Even if useful under some circumstances, environmental treatments are primarily directed to the control of-host lifecycle stages of ectoparasites, which are resting in hidden and inaccessible places most of the time [24,25]. This, in our case, was amplified by the particular surroundings of the refuge that was constructed in an old citrus plantation abounding in ideal sites for off-host stages (e.g. limestone walls, trees, bushes and several cracks and crevices on walls and ground). Under these conditions, the high efficacy of imidacloprid/flumethrin slow release collar in fast curing pre-existing ectoparasite infestations and preventing new ones is explained by the particular key features associated with this new product that makes it a reliable tool for the control of ectoparasites in dogs. Indeed, the two active molecules in the collar exhibit a neuro-pharmacological excitatory effect through a synergistic mechanism [12,13,26]. In addition, the polymer matrix system ensures that both active ingredients are slowly and continuously released from the collar towards the animal, thereby avoiding peak concentrations and ensuring that concentrations are present in the animal hair coat during the entire efficacy period [13]. This unique association of technology, long-term efficacy and ease of use have contributed to the excellent results observed in our investigation. The contemporaneous application of collars to almost all the dogs hosted in the shelter has produced a fast killing effect of the pre-existing parasitic stages on dogs (92.5% in 48 h for ticks) and the complete removal of any ectoparasite infestation from treated animals within 14 days. Collars also proved to be efficacious in preventing new infestations. Crawling ectoparasites were, indeed, soon repelled from collared animals (Additional file 1) and no more ticks and/or fleas were found on dogs after day 14, excluding one minimally tick infested and two flea infested dogs at day 30, and two tick infested dogs at day 250. In our investigation no negative control group was planned for ethical reasons. However, in five dogs, allowed to free range inside the refuge premises, it was not possible to place collars due to their scared and unfriendly behaviour. Interestingly, in these dogs evident tick infestations were regularly observed from day 0 up to day 60 which confirmed, at least for this period, the constant risk for dogs housed in the refuge of being infested by ticks (Figure 6). Cases of FAD represent another indirect way for the evaluation of efficacy against ectoparasites, in this case fleas. It is well known that animals suffering from FAD can easily show relapses especially when treated with short-term duration insecticides (e.g. spot-on) when the inter-treatment period is inappropriately stretched [13,27,28]. It is important to note that in our investigation all the 10 cases of pre-existing FAD completely resolved without the help of any palliative treatments within 30 days from collar application. This result confirms the high and long-term efficacy against flea infestation of the investigated collars and suggests how these can be effectively employed as a unique strategy for the treatment and prevention of FAD in dogs. With regards to the safety of the employed product, with the exception of one case of a local and transient reaction at the collar application site no other suspected adverse drug reaction was observed in treated animals. Furthermore, none of the enrolled dogs lost the collar during the period of investigation. The case of local reaction at the collar site was characterized by an erythematous dermatitis on the ventral side of the neck that appeared about 45 days after application of the collar and healed spontaneously without any treatments when the collar was removed. It is likely that this reaction was due to mechanical rubbing of the collar, which might have been fitted too tightly and/or for a particular skin sensitivity of the treated dog. These observations of overall high safety reflect the experiences made on the product’s safety and toxicity in previous studies [12,13]. They confirm that this device can be considered safe also in a particular setting such as refuges or dog communities where dogs are mostly housed in collective pens and allowed to freely range around trees and bushes. Finally, another result of great value was produced on shelter volunteers who, comprehensibly, appreciated the interruption of the biocide sprayings that had little effect, allowing notable savings of both money and time Volunteers appreciated the fast resolution of infestations and were no longer afraid of being bitten by ticks or fleas, and felt comfortable again when accomplishing their activities with dogs and also when they were inside the pens.

## Conclusions

Ectoparasites of dogs represent a year-round hazard and are of great importance in veterinary medicine. In some particular settings such as dog refuges, sustainable and long-term strategies to control ectoparasite infestations are needed. Environmental treatments as well as topical treatments (e.g. spot-on or sprays) entail main disadvantages that considerably limit their use and efficacy in large dog communities. Based on the observed evidence of efficacy, long-term duration and safety the imidacloprid/flumethrin slow release collars can be regarded as an efficacious and sustainable means for ectoparasite control and for treatment of FAD in high-risk dog communities.

## Competing interests

The authors declare that they have no competing interests.

## Authors’ contributions

EB and LF conceived the research, collected data in the field, wrote the first draft, contributed with data analysis and interpretation and revised the manuscript. GG, EN and SG supported the collection of data in the field and in the laboratory, contributed with data analysis and revision of the manuscript. CP contributed with interpretation and revision of the manuscript. All authors read and approved the final version of the manuscript.

## Supplementary Material

Additional file 1 **Tick repelled from a collared dog.** Movie shows a tick that is soon repelled from the treated animal with evident neurological excitatory effect caused by the two active molecules contained in the slow release collar. Click here for file

## References

[B1] Beugnet F Mariè JL Emerging arthropod-borne diseases of companion animals in Europe Vet Parasitol 2009 163 298 305 10.1016/j.vetpar.2009.03.028 19403239

[B2] Otranto D Dantas-Torres F Canine and feline vector-borne diseases in Italy: current situation and perspectives Parasit Vectors 2010 3 2 10.1186/1756-3305-3-2 20145730 PMC2818618

[B3] Chomel B Tick-borne infections in dogs-an emerging infectious threat Vet Parasitol 2011 179 294 301 10.1016/j.vetpar.2011.03.040 21777730

[B4] Dantas-Torres F Chomel BB Otranto D Ticks and tick-borne diseases: a One health perspective Trends Parasitol 2012 2 437 446 22902521 10.1016/j.pt.2012.07.003

[B5] Lorusso V Dantas-Torres F Lia RP Tarallo VD Mencke N Capelli G Otranto D Seasonal dynamics of the brown dog tick, *Rhipicephalus sanguineus*, on a confined dog population in Italy Med Vet Entomol 2010 24 309 315 20557458 10.1111/j.1365-2915.2010.00885.x

[B6] World Organisation for Animal Health Stray dog population control Terrestrial Animal Health Code 2010 Paris: OIE 403 419

[B7] Voslářvá E Passantino A Stray dog and cat laws and enforcement in Czech Republic and in Italy Ann Ist Super Sanita 2012 48 97 104 22456023 10.4415/ANN_12_01_16

[B8] Brianti E Pennisi MG Brucato G Risitano AL Gaglio G Lombardo G Malara D Fogliazza A Giannetto S Efficacy of the fipronil 10%+(S)-methoprene 9% combination against *Rhipicephalus sanguineus* in naturally infested dogs: speed of kill, persistent efficacy on immature and adult stages and effect of water Vet Parasitol 2010 170 96 103 10.1016/j.vetpar.2010.01.033 20185241

[B9] Otranto D Wall R New strategies for the control of arthropod vectors of disease in dogs and cats Med Vet Entomol 2008 22 291 302 10.1111/j.1365-2915.2008.00741.x 18785935

[B10] Casida JE Durkin KA Neuroactive insecticides: targets, selectivity, resistance, and secondary effects Annu Rev Entomol 2013 58 99 117 10.1146/annurev-ento-120811-153645 23317040

[B11] Radakovic M Stevanovic J Djelic N Lakic N Knezevic-Vukcevic J Vukovic-Gacic B Stanimirovic Z Evaluation of the DNA damaging effects of amitraz on human lymphocytes in the comet assay J Biosci 2013 38 53 62 10.1007/s12038-012-9287-2 23385813

[B12] Stanneck D Ebbinghaus-Kintscher U Schoenhense E Kruedewagen EM Turberg A Leisewitz A Jiritschka W Krieger KJ The synergistic action of imidacloprid and flumethrin and their release kinetics from collars applied for ectoparasite control in dogs and cats Parasit Vectors 2012 5 73 10.1186/1756-3305-5-73 22498105 PMC3361670

[B13] Stanneck D Rass J Radeloff I Kruedewagen E Le Sueur C Hellmann K Krieger K Evaluation of the long-term efficacy and safety of an imidacloprid 10%/flumethrin 4.5% polymer matrix collar (Seresto®) in dogs and cats naturally infested with fleas and/or ticks in multicentre clinical field studies in Europe Parasit Vectors 2012 5 66 10.1186/1756-3305-5-66 22463745 PMC3353155

[B14] Otranto D Dantas-Torres F de Capraris D Di Paola G Tarallo VD Latrofa MS Lia RP Annoscia G Breitshwerdt EB Cantacessi C Capelli G Stanneck D Prevention of canine leishmaniosis in a hyper-endemic area using a combination of 10% imidacloprid/4.5% flumethrin Plos One 2013 8 2 10.1371/journal.pone.0056374 PMC3581506 23451043

[B15] Manilla G Fauna d'Italia Vol XXXVI Acari-Ixodida 1998 Bologna: Edizioni Calderini

[B16] Berlinguer G Aphaniptera d’Italia: studio monografico 1964 Roma: Il Pensiero Scientifico

[B17] Dantas-Torres F Latrofa MS Annoscia G Giannelli A Parisi A Otranto D Morphological and genetic diversity of *Rhipicephalus sanguineus sensu lato* from the New and Old Worlds Parasit Vectors 2013 in press 10.1186/1756-3305-6-213 PMC3735430 23880226

[B18] Bush AO Lafferty KD Lotz JM Shostak AW Parasitology meets ecology on its own terms: Margolis et al. revisited J Parasitol 1997 83 575 583 10.2307/3284227 9267395

[B19] Otranto D Brianti E Abramo F Gaglio G Napoli E Latrofa MS Ramos RA Torres F Bain O Cutaneous distribution and localization of *Cercopithifilaria* sp. microfilariae in dogs Vet Parasitol 2012 190 143 150 10.1016/j.vetpar.2012.05.016 22698796

[B20] Koch HG Seasonal incidence and attachment sites of ticks (Acari: Ixodidae) on domestic dogs in southeastern Oklahoma and northwestern Arkansas, USA J Med Entomol 1982 19 293 298 7120309 10.1093/jmedent/19.3.293

[B21] Tinoco-Gracia L Quiroz-Romero H Quintero-Martínez MT Rentería-Evangelista TB González-Medina Y Barreras-Serrano A Hori-Oshima S Moro MH Vinasco J Prevalence of *Rhipicephalus sanguineus* ticks on dogs in a region on the Mexico-USA border Vet Rec 2009 164 59 61 10.1136/vr.164.2.59 19136688

[B22] Silveira JA Passos LM Ribeiro MF Population dynamics of *Rhipicephalus sanguineus* (Latrielle, 1806) in Belo Horizonte, Minas Gerais state, Brazil Vet Parasitol 2009 161 270 275 10.1016/j.vetpar.2009.01.028 19339119

[B23] Duscher GG Feiler A Leschnik M Joachim A Seasonal and spatial distribution of ixodid tick species feeding on naturally infested dogs from Eastern Austria and the influence of acaricides/repellents on these parameters Parasit Vectors 2013 6 76 10.1186/1756-3305-6-76 23510263 PMC3621693

[B24] Rust MK Advances in the control of *Ctenocephalides felis* (cat flea) on cats and dogs Trends Parasitol 2005 21 232 236 10.1016/j.pt.2005.03.010 15837612

[B25] Dantas-Torres F Biology and ecology of the brown dog tick Rhipicephalus sanguineus. Parasit Vectors 2010 3 26 10.1186/1756-3305-3-26 PMC2857863 20377860

[B26] Stanneck D Kruedewagen EM Fourie JJ Horak IG Davis W Krieger KJ Efficacy of an imidacloprid/flumethrin collar against fleas, ticks, mites and lice on dogs Parasit Vectors 2012 5 102 10.1186/1756-3305-5-102 22647530 PMC3433312

[B27] Dryden MW Flea and tick control in the 21st century: challenges and opportunities Vet Dermatol 2009 20 435 440 10.1111/j.1365-3164.2009.00838.x 20178481

[B28] Overgaauw P Frontline Combo sucht die Schutzpraxis Kleintierpraxis 2011 56 504

